# Supplementary material for: Indigenous cattle of Sri Lanka: Genetic and phylogeographic relationship with Zebu of Indus Valley and South Indian origin
Source: PLoS One. 2023 Aug 16;18(8):e0282761. doi: 10.1371/journal.pone.0282761 (PMC10431622; doi:10.1371/journal.pone.0282761)
Supplement: S1 File — (DOCX) [file pone.0282761.s001.docx]

S1 file. Global F statistics among Sri Lankan, Indus Valley, South Indian Zebu and commercial taurine cattle

| Locus | All cattle breeds | | | Zebu cattle only | | |
| --- | --- | --- | --- | --- | --- | --- |
|  | F_IT_ | F_ST_ | F_IS_ | F_IT_ | F_ST_ | F_IS_ |
| CSRM60 | 0.168±0.037 | 0.118±0.030 | 0.056±0.028 | 0.118±0.040 | 0.046±0.011 | 0.075±0.033 |
| CSSM66 | 0.106±0.034 | 0.122±0.028 | -0.018±0.025 | 0.024±0.025 | 0.051±0.016 | -0.028±0.036 |
| HEL1 | 0.205±0.048 | 0.217±0.044 | -0.016±0.041 | 0.075±0.040 | 0.082±0.024 | -0.006±0.048 |
| INRA63 | 0.208±0.048 | 0.205±0.048 | 0.005±0.038 | 0.066±0.049 | 0.038±0.012 | 0.029±0.053 |
| BM1824 | 0.108±0.036 | 0.078±0.016 | 0.033±0.029 | 0.038±0.048 | 0.043±0.026 | -0.006±0.037 |
| HAUT27 | 0.313±0.055 | 0.115±0.043 | 0.223±0.050 | 0.357±0.077 | 0.084±0.068 | 0.297±0.048 |
| INRA05 | 0.103±0.032 | 0.099±0.020 | 0.004±0.031 | 0.055±0.023 | 0.053±0.016 | 0.002±0.031 |
| BM1818 | 0.162±0.033 | 0.099±0.025 | 0.070±0.036 | 0.140±0.044 | 0.044±0.016 | 0.101±0.041 |
| ETH3 | 0.208±0.037 | 0.183±0.042 | 0.032±0.036 | 0.130±0.051 | 0.067±0.046 | 0.067±0.023 |
| HEL9 | 0.171±0.049 | 0.128±0.039 | 0.050±0.044 | 0.158±0.050 | 0.080±0.030 | 0.086±0.054 |
| ILSTS006 | 0.130±0.053 | 0.093±0.024 | 0.042±0.054 | 0.103±0.082 | 0.035±0.011 | 0.070±0.080 |
| HAUT24 | 0.285±0.038 | 0.141±0.026 | 0.168±0.050 | 0.328±0.039 | 0.101±0.030 | 0.253±0.043 |
| HEL5 | 0.496±0.046 | 0.279±0.059 | 0.303±0.059 | 0.506±0.041 | 0.159±0.079 | 0.415±0.053 |
| INRA032 | 0.213±0.039 | 0.169±0.044 | 0.054±0.027 | 0.118±0.017 | 0.065±0.027 | 0.057±0.019 |
| SPS115 | 0.121±0.033 | 0.082±0.030 | 0.044±0.030 | 0.055±0.030 | 0.035±0.014 | 0.021±0.028 |
| ETH185 | 0.179±0.036 | 0.143±0.032 | 0.042±0.024 | 0.117±0.025 | 0.072±0.021 | 0.049±0.030 |
| HEL13 | 0.266±0.082 | 0.268±0.066 | -0.003±0.051 | -0.003±0.067 | 0.023±0.018 | -0.027±0.070 |
| ILSTS05 | 0.143±0.039 | 0.131±0.032 | 0.014±0.035 | 0.077±0.047 | 0.049±0.024 | 0.029±0.041 |
| INRA035 | 0.328±0.061 | 0.187±0.055 | 0.174±0.060 | 0.221±0.031 | 0.127±0.041 | 0.109±0.053 |
| TGLA126 | 0.085±0.029 | 0.106±0.017 | -0.023±0.027 | 0.022±0.019 | 0.071±0.015 | -0.053±0.023 |
| BM2113 | 0.242±0.051 | 0.116±0.037 | 0.142±0.042 | 0.201±0.054 | 0.062±0.031 | 0.148±0.052 |
| ETH10 | 0.154±0.039 | 0.163±0.033 | -0.011±0.028 | 0.072±0.059 | 0.091±0.055 | -0.020±0.038 |
| ETH225 | 0.213±0.062 | 0.199±0.053 | 0.017±0.027 | 0.056±0.050 | 0.057±0.037 | -0.000±0.031 |
| INRA023 | 0.152±0.042 | 0.131±0.033 | 0.024±0.026 | 0.062±0.040 | 0.064±0.028 | -0.002±0.026 |
| TGLA122 | 0.058±0.028 | 0.089±0.020 | -0.034±0.022 | 0.039±0.033 | 0.051±0.015 | -0.012±0.026 |
| Overall | 0.190±0.018 | 0.145±0.010 | 0.054±0.016 | 0.124±0.022 | 0.065±0.005 | 0.063±0.020 |
