# Supplementary material for: Indigenous cattle of Sri Lanka: Genetic and phylogeographic relationship with Zebu of Indus Valley and South Indian origin
Source: PLoS One. 2023 Aug 16;18(8):e0282761. doi: 10.1371/journal.pone.0282761 (PMC10431622; doi:10.1371/journal.pone.0282761)

S2 file. The test for selective neutrality at 27 microsatellite marker loci. Selection detection based on the *F*_ST_ outlier approach using LOSITAN under the assumption of infinite allele mutation model


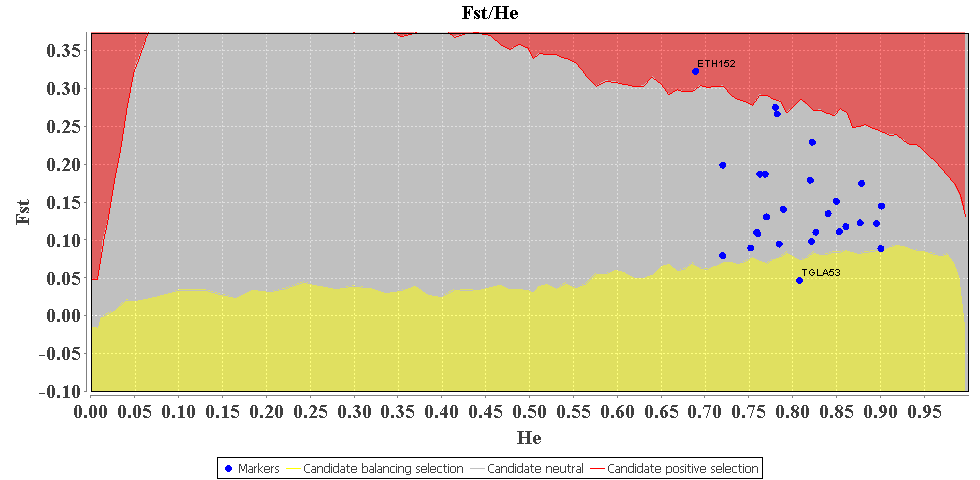

Supplement: S2 File — Selection detection based on the FST outlier approach using LOSITAN under the assumption of infinite allele mutation model. (DOCX) [file pone.0282761.s002.docx]
