# Supplementary material for: Indigenous cattle of Sri Lanka: Genetic and phylogeographic relationship with Zebu of Indus Valley and South Indian origin
Source: PLoS One. 2023 Aug 16;18(8):e0282761. doi: 10.1371/journal.pone.0282761 (PMC10431622; doi:10.1371/journal.pone.0282761)

S3 file. Determination of correct number of clusters in Bayesian STRUCTURE analysis (Evanno et al. 2005) (a) Mean L (K) over 20 runs for each K value of 1 to 14 (b) Distribution of ∆K with the modal value (K=2) indicating the true K or the uppermost level of structure


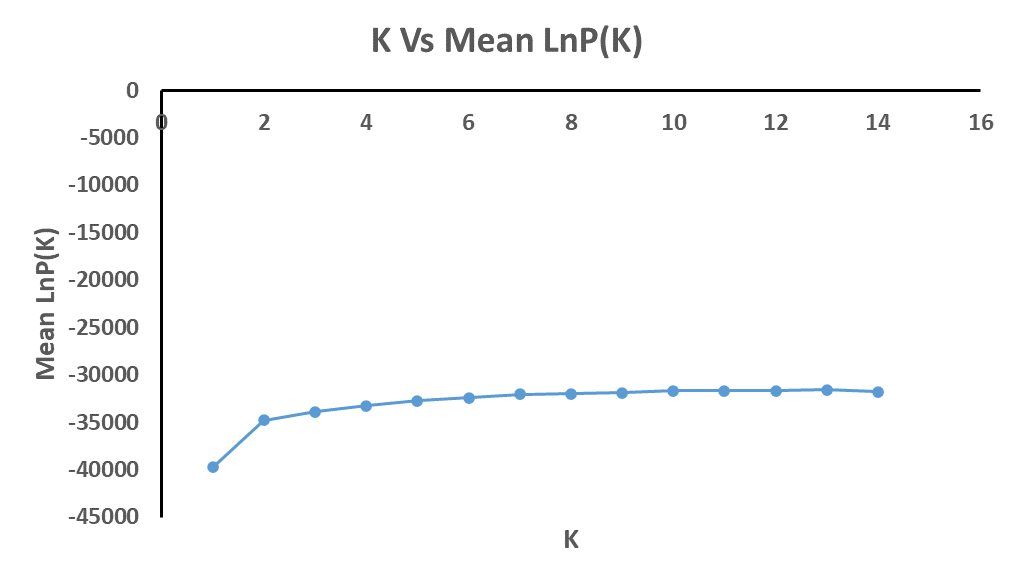


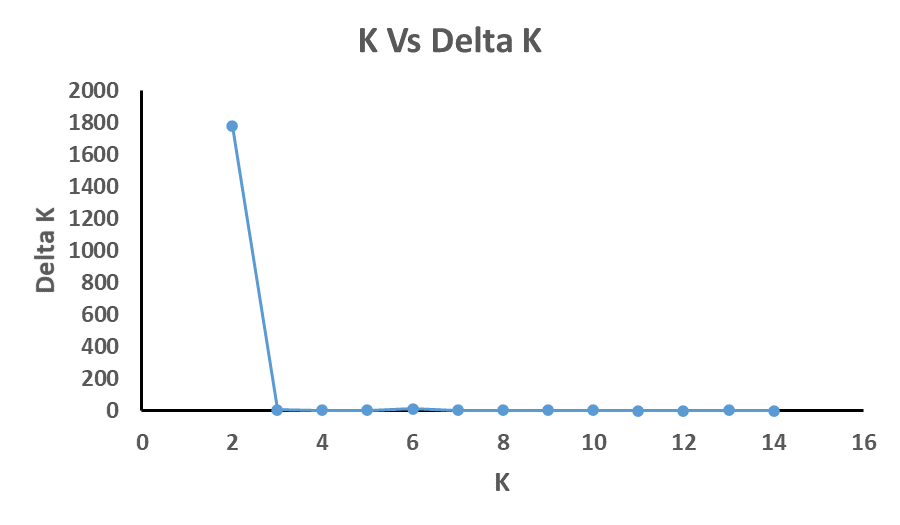

Supplement: S3 File — 2005) (a) Mean L (K) over 20 runs for each K value of 1 to 14 (b) Distribution of ΔK with the modal value (K = 2) indicating the true K or the uppermost level of structure. (DOCX) [file pone.0282761.s003.docx]
