# Supplementary material for: Indigenous cattle of Sri Lanka: Genetic and phylogeographic relationship with Zebu of Indus Valley and South Indian origin
Source: PLoS One. 2023 Aug 16;18(8):e0282761. doi: 10.1371/journal.pone.0282761 (PMC10431622; doi:10.1371/journal.pone.0282761)
Supplement: S4 File — (DOCX) [file pone.0282761.s004.docx]

S4 file. Frequency of mtDNA haplotypes and their sharing among different breeds of Sri Lankan, Indus valley and South Indian zebu

| Haplotype Name | LBH | LWC | LTM | PRS | PSH | PTP | IHL | IKA |
| --- | --- | --- | --- | --- | --- | --- | --- | --- |
| B_H1_I1 | 0,039 | - | - | - | - | - | - | - |
| B_H2_I1 | 0,020 | - | - | - | - | - | - | - |
| B_H3_I1 | 0,118 | - | - | - | - | - | - | - |
| B_H4_I1 | 0,020 | - | - | - | - | - | - | - |
| B_H5_I1 | 0,020 | - | - | - | - | - | - | - |
| B_H6_I1 | 0,020 | - | - | - | - | - | - | - |
| B_H7_I1 | 0,020 | - | - | - | - | - | - | - |
| B_H8_I1 | 0,020 | - | - | - | - | - | - | - |
| B_H9_I1 | 0,059 | - | - | - | - | - | - | - |
| B_H10_I1 | 0,039 | - | - | - | - | - | - | - |
| B_H11_I1 | 0,039 | - | - | - | - | - | - | - |
| B_H12_I1 | 0,020 | - | - | - | - | - | - | - |
| B_H13_I1 | 0,020 | - | - | - | - | - | - | - |
| B_H14_I1 | 0,039 | - | - | - | - | - | - | - |
| B_H15_I1 | 0,020 | - | - | - | - | - | - | - |
| BW_H16_I1 | 0,020 | 0,039 | - | - | - | - | - | - |
| BW_H17_I1 | 0,020 | 0,098 | - | - | - | - | - | - |
| BWH_H18_I1 | 0,020 | 0,020 | - | - | - | - | 0,028 | - |
| BWRSPHK_H19_I1 | 0,118 | 0,412 | - | 0,138 | 0,276 | 0,296 | 0,139 | 0,216 |
| BWSHK_H20_I1 | 0,020 | 0,020 | - | - | 0,069 | - | 0,083 | 0,020 |
| H_H21_I1 | - | - | - | - | - | - | 0,028 | - |
| H_H22_I1 | - | - | - | - | - | - | 0,056 | - |
| H_H23_I1 | - | - | - | - | - | - | 0,028 | - |
| H_H24_I1 | - | - | - | - | - | - | 0,028 | - |
| H_H25_I1 | - | - | - | - | - | - | 0,028 | - |
| H_H26_I1 | - | - | - | - | - | - | 0,028 | - |
| H_H27_I1 | - | - | - | - | - | - | 0,028 | - |
| H_H28_I1 | - | - | - | - | - | - | 0,028 | - |
| H_H29_I1 | - | - | - | - | - | - | 0,028 | - |
| H_H30_I1 | - | - | - | - | - | - | 0,028 | - |
| H_H31_I1 | - | - | - | - | - | - | 0,028 | - |
| K_H32_I1 | - | - | - | - | - | - | - | 0,020 |
| K_H33_I1 | - | - | - | - | - | - | - | 0,020 |
| K_H34_I1 | - | - | - | - | - | - | - | 0,039 |
| K_H35_I1 | - | - | - | - | - | - | - | 0,020 |
| K_H36_I1 | - | - | - | - | - | - | - | 0,020 |
| K_H37_I1 | - | - | - | - | - | - | - | 0,020 |
| P_H38_I1 | - | - | - | - | - | 0,074 | - | - |
| P_H39_I1 | - | - | - | - | - | 0,037 | - | - |
| P_H40_I1 | - | - | - | - | - | 0,037 | - | - |
| P_H41_I1 | - | - | - | - | - | 0,037 | - | - |
| P_H42_I1 | - | - | - | - | - | 0,111 | - | - |
| P_H43_I1 | - | - | - | - | - | 0,037 | - | - |
| P_H44_I1 | - | - | - | - | - | 0,074 | - | - |
| R_H45_I1 | - | - | - | 0,035 | - | - | - | - |
| R_H46_I1 | - | - | - | 0,035 | - | - | - | - |
| R_H47_I1 | - | - | - | 0,069 | - | - | - | - |
| R_H48_I1 | - | - | - | 0,035 | - | - | - | - |
| R_H49_I1 | - | - | - | 0,035 | - | - | - | - |
| R_H50_I1 | - | - | - | 0,035 | - | - | - | - |
| R_H51_I1 | - | - | - | 0,035 | - | - | - | - |
| R_H52_I1 | - | - | - | 0,035 | - | - | - | - |
| RH_H53_I1 | - | - | - | 0,069 | - | - | 0,028 | - |
| RSPK_H54_I1 | - | - | - | 0,035 | 0,035 | 0,074 | - | 0,039 |
| S_H55_I1 | - | - | - | - | 0,035 | - | - | - |
| S_H56_I1 | - | - | - | - | 0,035 | - | - | - |
| S_H57_I1 | - | - | - | - | 0,035 | - | - | - |
| S_H58_I1 | - | - | - | - | 0,035 | - | - | - |
| S_H59_I1 | - | - | - | - | 0,069 | - | - | - |
| S_H60_I1 | - | - | - | - | 0,035 | - | - | - |
| T_H61_I1 | - | - | 0,200 | - | - | - | - | - |
| T_H62_I1 | - | - | 0,040 | - | - | - | - | - |
| TRS_H63_I1 | - | - | 0,160 | 0,035 | 0,035 | - | - | - |
| TSK_H64_I1 | - | - | 0,360 |  | 0,035 | - | - | 0,039 |
| W_H65_I1 | - | 0,059 | - | - | - | - | - | - |
| W_H66_I1 | - | 0,020 | - | - | - | - | - | - |
| W_H67_I1 | - | 0,020 | - | - | - | - | - | - |
| W_H68_I1 | - | 0,020 | - | - | - | - | - | - |
| WK_H69_I1 | - | 0,039 | - | - | - | - | - | 0,255 |
| B_H70_I2 | 0,078 | - | - | - | - | - | - | - |
| B_H71_I2 | 0,020 | - | - | - | - | - | - | - |
| B_H72_I2 | 0,098 | - | - | - | - | - | - | - |
| B_H73_I2 | 0,020 | - | - | - | - | - | - | - |
| BHK_H74_I2 | 0,020 | - | - | - | - | - | 0,056 | 0,020 |
| BT_H75_I2 | 0,020 | - | 0,080 | - | - | - | - | - |
| H_H76_I2 | - | - | - | - | - | - | 0,028 | - |
| H_H77_I2 | - | - | - | - | - | - | 0,028 | - |
| H_H78_I2 | - | - | - | - | - | - | 0,028 | - |
| H_H79_I2 | - | - | - | - | - | - | 0,028 | - |
| H_H80_I2 | - | - | - | - | - | - | 0,028 | - |
| H_H81_I2 | - | - | - | - | - | - | 0,028 | - |
| H_H82_I2 | - | - | - | - | - | - | 0,028 | - |
| H_H83_I2 | - | - | - | - | - | - | 0,028 | - |
| K_H84_I2 | - | - | - | - | - | - | - | 0,059 |
| K_H85_I2 | - | - | - | - | - | - | - | 0,020 |
| K_H86_I2 | - | - | - | - | - | - | - | 0,020 |
| P_H87_I2 | - | - | - | - | - | 0,074 | - | - |
| R_H88_I2 | - | - | - | 0,035 | - | - | - | - |
| R_H89_I2 | - | - | - | 0,035 | - | - | - | - |
| R_H90_I2 | - | - | - | 0,035 | - | - | - | - |
| R_H91_I2 | - | - | - | 0,069 | - | - | - | - |
| R_H92_I2 | - | - | - | 0,035 | - | - | - | - |
| R_H93_I2 | - | - | - | 0,035 | - | - | - | - |
| R_H94_I2 | - | - | - | 0,069 | - | - | - | - |
| R_H95_I2 | - | - | - | 0,035 | - | - | - | - |
| S_H96_I2 | - | - | - | - | 0,035 | - | - | - |
| S_H97_I2 | - | - | - | - | 0,035 | - | - | - |
| S_H98_I2 | - | - | - | - | 0,035 | - | - | - |
| SH_H99_I2 | - | - | - | - | 0,035 | - | 0,028 |  |
| SP_H100_I2 | - | - | - | - | 0,035 | 0,037 | - | - |
| W_H101_I2 | - | 0,020 | - | - | - | - | - | - |
| W_H102_I2 | - | 0,020 | - | - | - | - | - | - |
| W_H103_I2 | - | 0,020 | - | - | - | - | - | - |
| W_H104_I2 | - | 0,020 | - | - | - | - | - | - |
| WRS_H105_I2 | - | 0,020 | - | 0,035 | 0,035 | - | - | - |
| WTRPHK_H106_I2 | - | 0,157 | 0,160 | 0,035 |  | 0,111 | 0,056 | 0,157 |
| H_H107_T2 | - | - | - | - | - | - | 0,028 | - |
| K_H108_T2 | - | - | - | - | - | - | - | 0,020 |
| B_H109_T3 | 0,020 | - | - | - | - | - | - | - |
| B_H110_T3 | 0,020 | - | - | - | - | - | - | - |
| S_H111_T3 | - | - | - | - | 0,069 | - | - | - |
| S_H112_T3 | - | - | - | - | 0,035 | - | - | - |
