# Supplementary material for: Indigenous cattle of Sri Lanka: Genetic and phylogeographic relationship with Zebu of Indus Valley and South Indian origin
Source: PLoS One. 2023 Aug 16;18(8):e0282761. doi: 10.1371/journal.pone.0282761 (PMC10431622; doi:10.1371/journal.pone.0282761)

S5 file. Maximum likelihood tree of mitochondrial DNA haplotypes of Sri Lankan, Indus valley and South Indian zebu (B-Batu Harak; W-Lanka White; T-Thawalam; R-Red Sindhi; S-Sahiwal; P-Tharparkar; H-Hallikar; K-Kangayam).


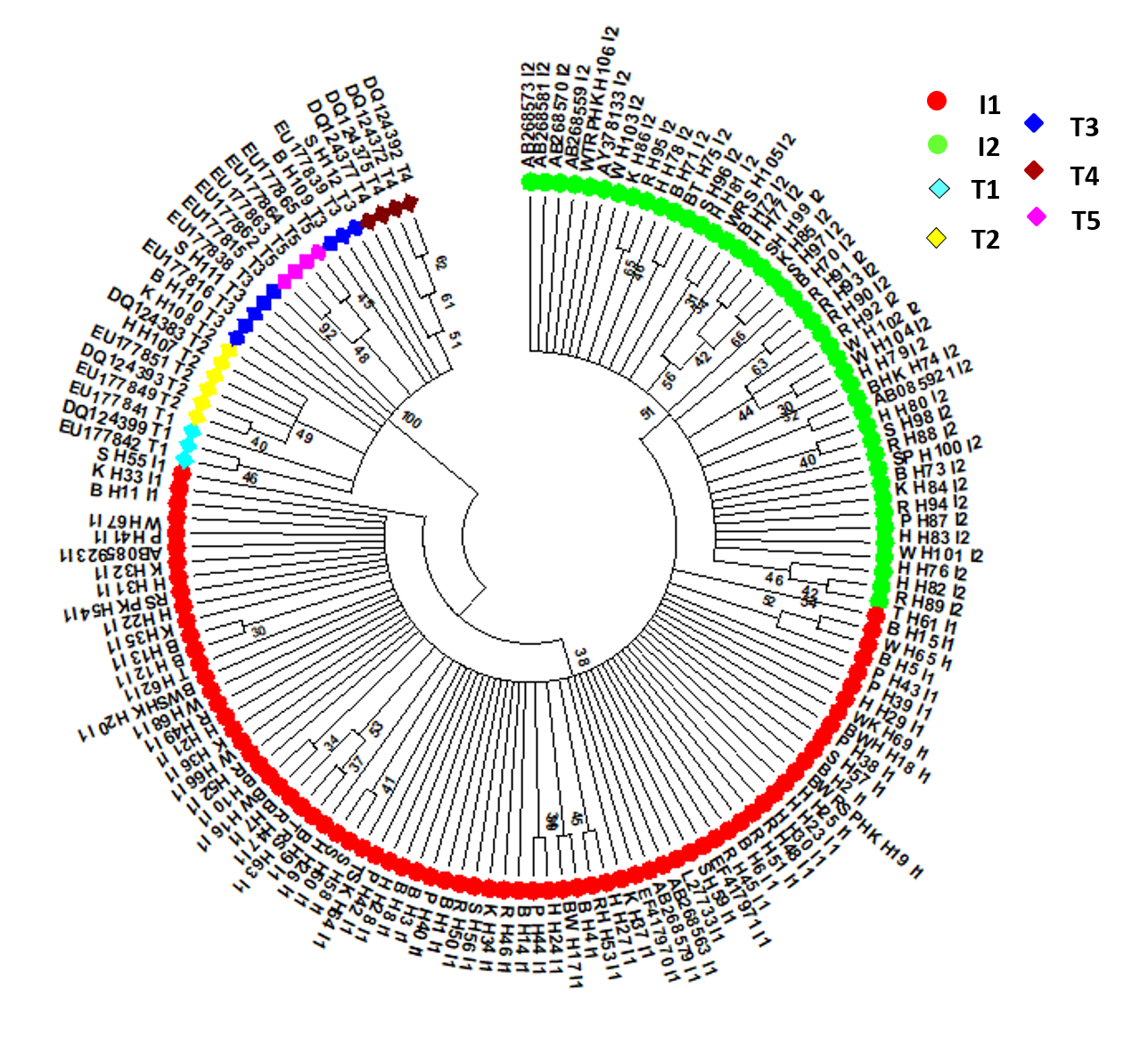

Supplement: S5 File — (DOCX) [file pone.0282761.s005.docx]
